# Supplementary material for: The airway microbiota of neonates colonized with asthma-associated pathogenic bacteria
Source: Nat Commun. 2023 Oct 20;14:6668. doi: 10.1038/s41467-023-42309-z (PMC10589220; doi:10.1038/s41467-023-42309-z)
Supplement: Supplementary file 1 — Supplementary Information [file 41467_2023_42309_MOESM1_ESM.pdf]

SUPPLEMENTARY INFORMATION

For the article Thorsen J et al, “The airway microbiota of neonates colonized with asthma-associated pathogenic bacteria”, *Nature Communications* 2023.

Table of Contents

Supplementary Fig. 1 2

Supplementary Fig. 2 3

Supplementary Fig. 3 4

Supplementary Fig. 4 5

Supplementary Fig. 5 6

Supplementary Fig. 6 8

Supplementary Table 1 9

Supplementary Table 2 10

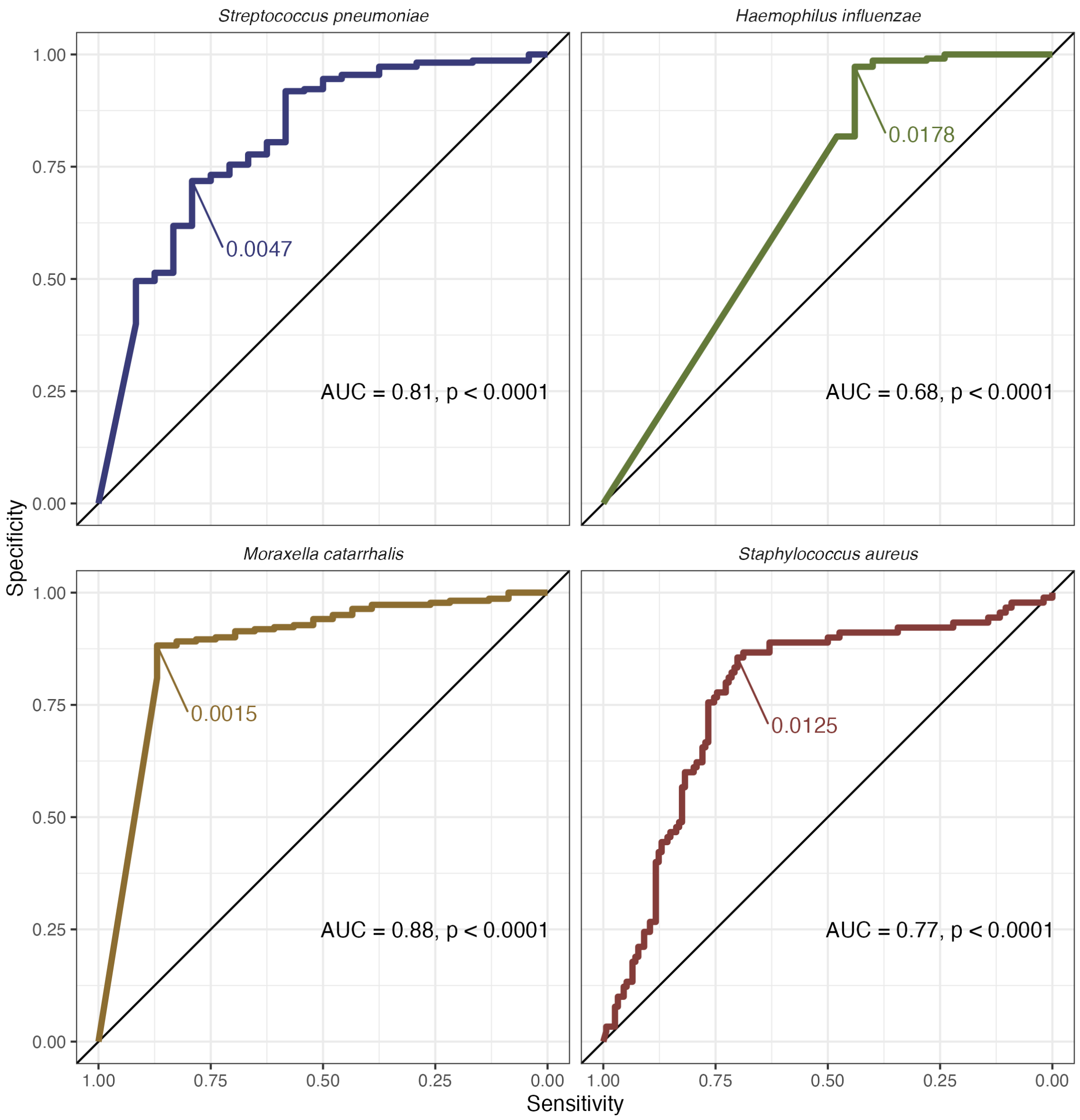

Supplementary Fig. 1

Receiver Operating Characteristic (ROC) curves for comparing relative abundances from nasopharynx swabs with culture results from hypopharynx aspirates, for the four cultured bacterial species. Area under the curve (AUC) values and p-values (Wilcoxon tests) are shown for each comparison. Cutoff values marked for each curve (relative abundance) corresponding to the maximum value of Youden's J index. N = 285 children.

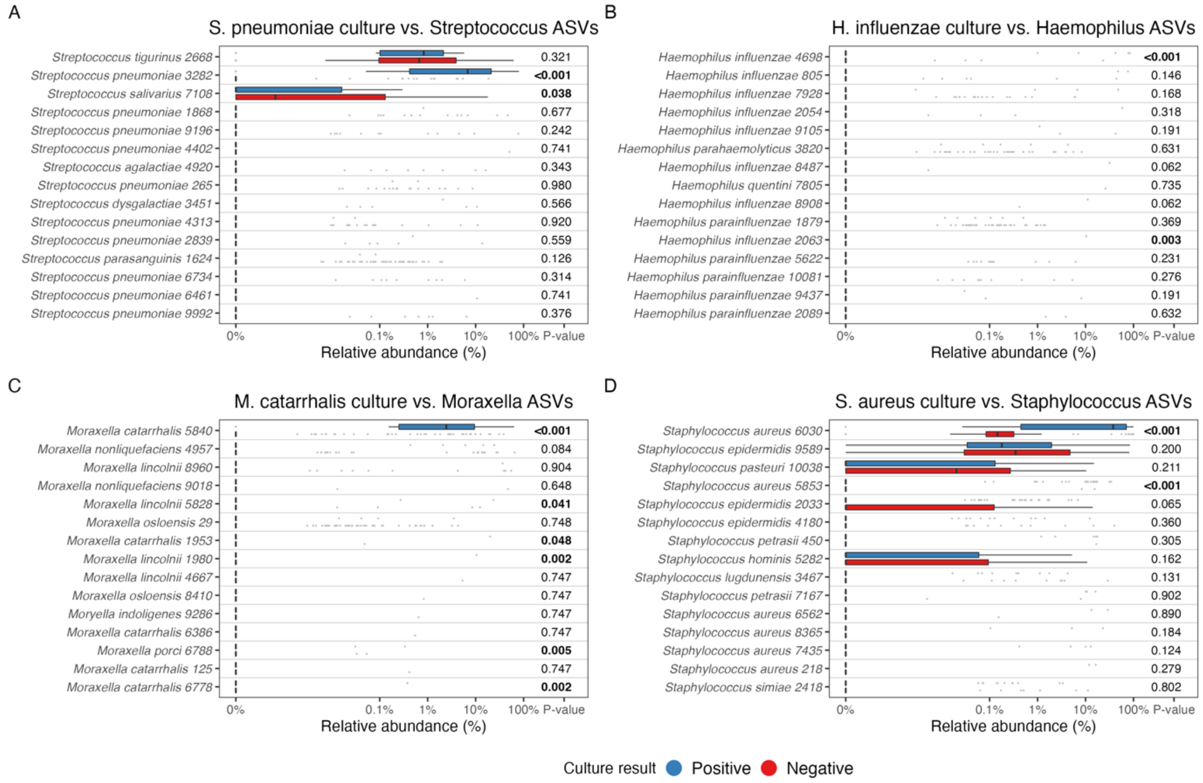

Supplementary Fig. 2

Comparison of the culture results (positive/negative) for each of the four species identified from the hypopharynx aspirates (Panel A – *Streptococcus pneumoniae*; B – *Haemophilus influenzae*; C – *Moraxella catarrhalis*; D – *Staphylococcus aureus*) and the relative abundances from 16S sequencing of nasopharyngeal swabs of each of the 15 most abundant Amplicon Sequence Variants (ASVs) within the same genus of each. The results show high specificity and agreement at species level. P-values are from Wilcoxon tests. Box plots show median (middle line), interquartile range (IQR, box), 1.5xIQR range (whiskers) and outliers (dots). A pseudocount of 1e-7 was added for visualization purposes, corresponding to “0%” on the x-axis. N=285.

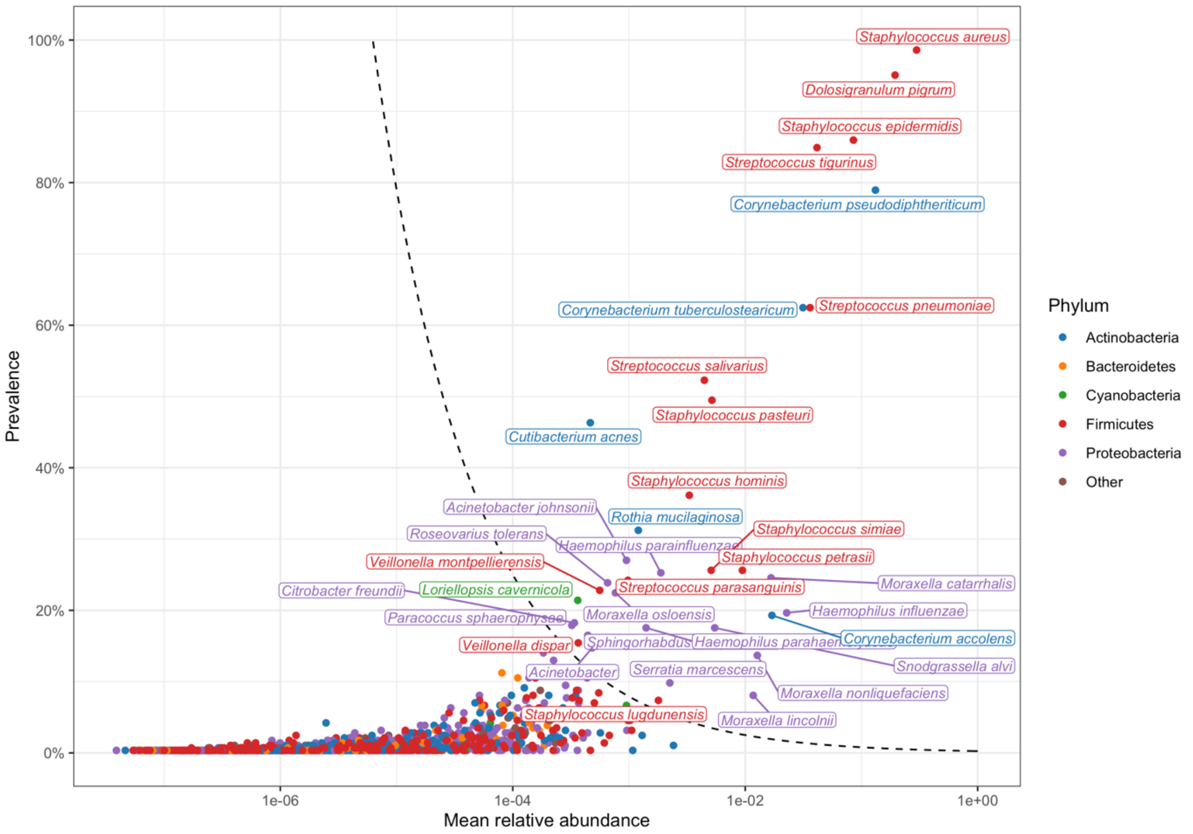

Supplementary Fig. 3

Comparison of mean relative abundance and prevalence for all species identified in the 16S sequencing data from nasopharyngeal swabs (n=285). The 35 most common species above the dashed line (Prevalence = 25%*(1e-4/Mean Relative Abundance)^0.5) are annotated and were selected for downstream analysis.

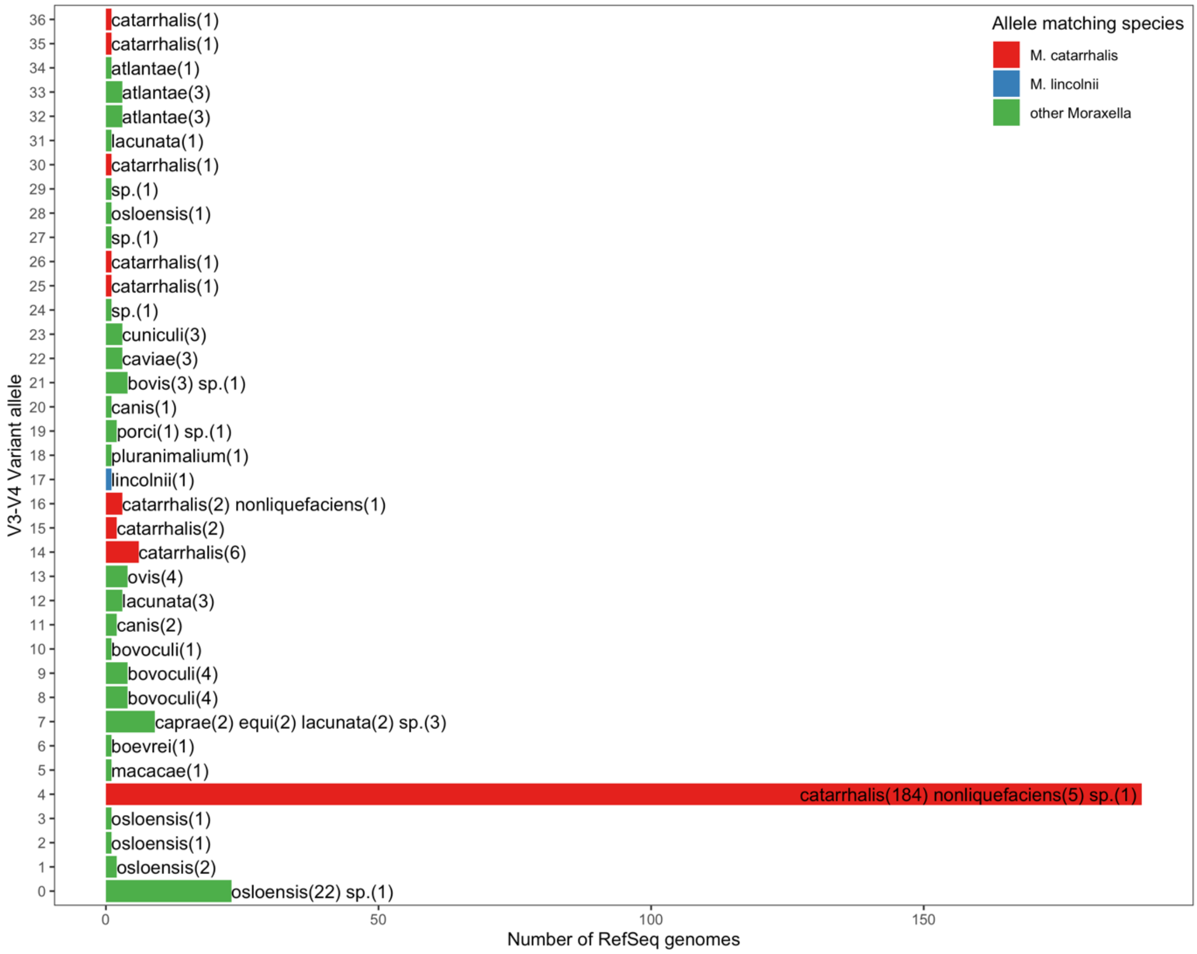

Supplementary Fig. 4

Comparison of simulated V3-V4 amplicons using the RibDif tool, based on RefSeq *Moraxella* genomes. While *M. catarrhalis* could be mistaken for *M. nonliquefaciens*, since their alleles are similar and cluster together, no other *Moraxella* species cluster with *M. lincolnii*.

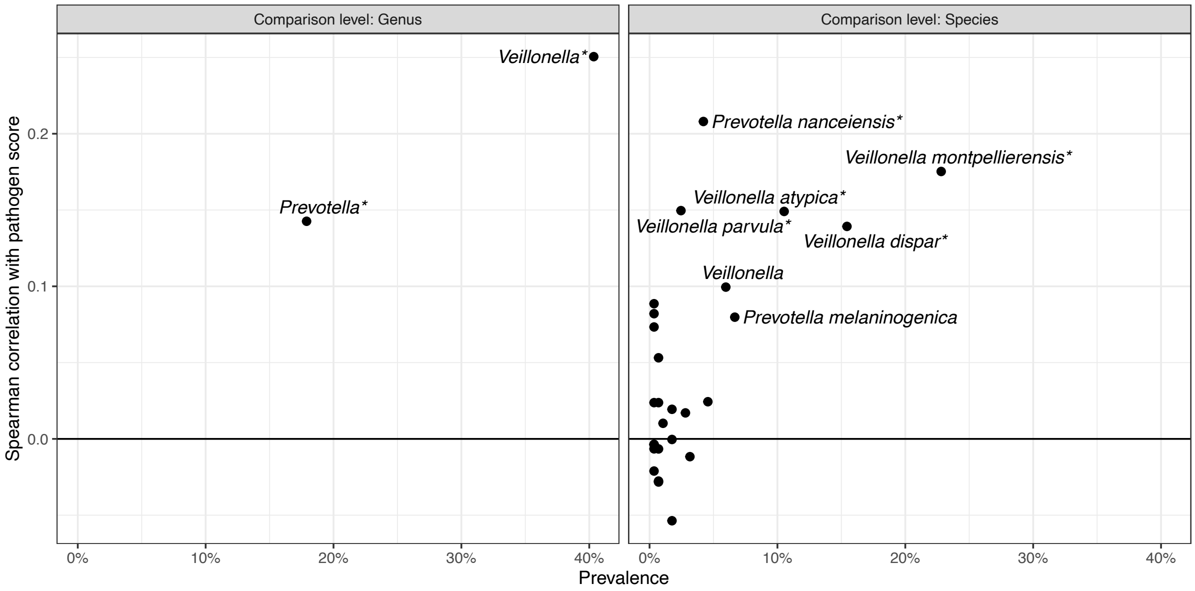

Supplementary Fig. 5

Spearman correlations between the pathogen score (derived from the a priori summed relative abundance of *S. pneumoniae, H. influenzae,* and *M. catarrhalis*) and the genera *Veillonella* and *Prevotella*. Significant correlations (p<0.05) are marked with *. Both *Veillonella*, *Prevotella* and several of their species constituents are significantly associated with the pathogen score. N=285.

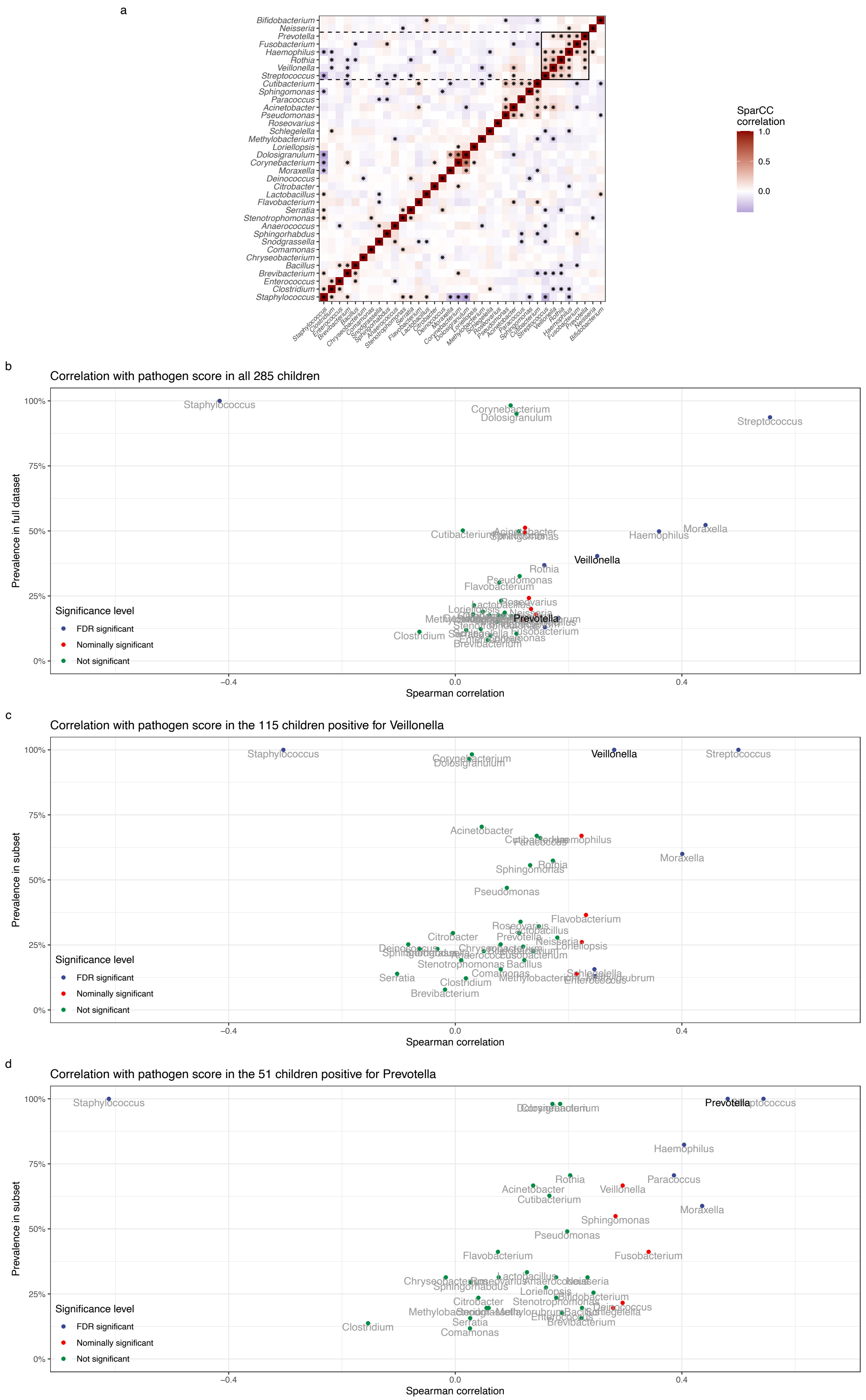

a
SparCC correlation
1.0
0.5
0.0
Bifidobacterium
Neisseria
Prevotella
Fusobacterium
Haemophilus
Rothia
Veillonella
Streptococcus
Cutibacterium
Sphingomonas
Paracoccus
Acinetobacter
Pseudomonas
Roseovarius
Schlegelella
Methylobacterium
Loriellopsis
Dolosigranulum
Corynebacterium
Moraxella
Deinococcus
Citrobacter
Lactobacillus
Flavobacterium
Serratia
Stenotrophomonas
Anaerococcus
Sphingorhabdus
Snodgrassella
Comamonas
Chryseobacterium
Bacillus
Brevibacterium
Enterococcus
Clostridium
Staphylococcus
b
Correlation with pathogen score in all 285 children
Prevalence in full dataset
Spearman correlation
Significance level
FDR significant
Nominally significant
Not significant
100%
75%
50%
25%
0%
−0.4
0.0
0.4
Staphylococcus
Corynebacterium
Dolosigranulum
Streptococcus
Moraxella
Haemophilus
Veillonella
Rothia
Pseudomonas
Flavobacterium
Prevotella
Clostridium
Brevibacterium
c
Correlation with pathogen score in the 115 children positive for Veillonella
Prevalence in subset
Spearman correlation
Significance level
FDR significant
Nominally significant
Not significant
Staphylococcus
Veillonella
Streptococcus
Acinetobacter
Moraxella
Pseudomonas
Flavobacterium
Citrobacter
Stenotrophomonas
Serratia
Brevibacterium
d
Correlation with pathogen score in the 51 children positive for Prevotella
Prevalence in subset
Spearman correlation
Significance level
FDR significant
Nominally significant
Not significant
Staphylococcus
Prevotella
Haemophilus
Rothia
Paracoccus
Acinetobacter
Veillonella
Cutibacterium
Moraxella
Sphingomonas
Pseudomonas
Flavobacterium
Fusobacterium
Clostridium
Citrobacter
Serratia
Comamonas
Brevibacterium

Supplementary Fig. 6

a) Correlation heatmaps between most common genera, arranged by hierarchical clustering, using the SparCC compositional correlation metric. Similar to fig 5, we observe that *Veillonella* and *Prevotella* form a cluster with two of the three pathogen genera, *Streptococcus* and *Haemophilus*, which also includes a few other taxa. Highlighting was performed manually. Stars indicate significant correlations (bootstrap p-values < 0.05). b-d) Correlations between the pathogen score and the most common genera in b) all children, c) children positive for *Veillonella* and d) children positive for *Prevotella*. In panel b), we observe that *Veillonella* has the fourth highest correlation, after the genera of the three pathogenic species on which the score was built. *Prevotella* is significant, but not after FDR correction. However, due to low prevalence and excess zeroes, the power to detect this correlation is low, which can be seen as a V-shape limit in the distribution of panel b. When subsetting the data to only those children positive for each genus, *Veillonella* remains strongly correlated and advances slightly to become the third most correlated, while *Prevotella* increases immensely to become the second most correlated, almost at the level of *Streptococcus*. N=285.

Predictor	N	Beta	95% CI[1]	p-value
Sex	285			0.2
Female		—	—	
Male		-0.16	-0.39, 0.07	
Ethnicity	285			0.5
Northern European		—	—	
Other		0.25	-0.42, 0.92	
Delivery mode	285			**0.009**
Vaginal		—	—	
Cesarean Section		-0.37	-0.65, -0.09	
Gestational age (weeks)	285	0.01	-0.06, 0.09	0.7
Birth weight (kg)	285	0.09	-0.13, 0.31	0.4
Birth length (cm)	285	0.02	-0.03, 0.07	0.3
Living environment	285			0.6
Rural		—	—	
Urban		-0.06	-0.29, 0.18	
Older siblings at home	267			**<0.001**
No siblings		—	—	
Siblings		0.63	0.39, 0.86	
Furred pet at home	271			0.6
No		—	—	
Yes		0.07	-0.19, 0.33	
Maternal smoking at 1 month	249			0.2
No		—	—	
Yes		0.21	-0.13, 0.55	
Breastfeeding at 1 month	257			0.091
No		—	—	
Yes		0.27	-0.04, 0.59	
Social circumstances PCA score	265	-0.02	-0.15, 0.10	0.7
Antibiotics during pregnancy	285			0.2
No antibiotics		—	—	
Antibiotics		0.16	-0.09, 0.42	

[1]CI = Confidence Interval

Supplementary Table 1

Key environmental factors and their association with the pathogen score, which was defined as the log-transformed and z-scaled combined relative abundances of *Streptococcus pneumoniae, Haemophilus influenzae,* and *Moraxella catarrhalis*. P-values are two-sided and not adjusted for multiple comparisons.

Genus	Species	Prevalence	N	Mean relative abundance	Genus HR for asthma COPSAC2010
Veillonella	*Veillonella montpellierensis*	22.81%	65	0.00056	1.45
Veillonella	*Veillonella dispar*	15.44%	44	0.000366	1.45
Veillonella	*Veillonella atypica*	10.53%	30	0.000157	1.45
Veillonella	*Veillonella sp.*	5.96%	17	0.000195	1.45
Veillonella	*Veillonella tobetsuensis*	4.56%	13	3.77E-05	1.45
Prevotella	*Prevotella melaninogenica*	6.67%	19	8.09E-05	1.32
Prevotella	*Prevotella nanceiensis*	4.21%	12	0.000139	1.32
Prevotella	*Prevotella veroralis*	3.16%	9	2.18E-05	1.32
Prevotella	*Prevotella jejuni*	1.75%	5	4.72E-06	1.32
Prevotella	*Prevotella histicola*	1.75%	5	2.81E-05	1.32
Gemella	*Gemella taiwanensis*	4.91%	14	6.56E-05	1.28
Gemella	*Gemella sanguinis*	2.46%	7	4.39E-05	1.28
Gemella	*Gemella parahaemolysans*	2.46%	7	4.14E-05	1.28
Gemella	*Gemella morbillorum*	2.11%	6	7.88E-06	1.28
Streptococcus	*Streptococcus tigurinus*	84.91%	242	0.041463	1.22
Streptococcus	*Streptococcus pneumoniae*	62.46%	178	0.036148	1.22
Streptococcus	*Streptococcus salivarius*	52.28%	149	0.00445	1.22
Streptococcus	*Streptococcus parasanguinis*	24.21%	69	0.000979	1.22
Streptococcus	*Streptococcus sanguinis*	5.96%	17	8.36E-05	1.22
Lactobacillus	*Lactobacillus curvatus*	7.37%	21	0.000371	1.21
Lactobacillus	*Lactobacillus gasseri*	6.32%	18	0.000168	1.21
Lactobacillus	*Lactobacillus iners*	3.16%	9	2.28E-05	1.21
Lactobacillus	*Lactobacillus sakei*	2.46%	7	9.83E-05	1.21
Lactobacillus	*Lactobacillus reuteri*	1.40%	4	3.89E-06	1.21
Neisseria	*Neisseria subflava*	7.37%	21	0.000129	1.18
Neisseria	*Neisseria cinerea*	7.02%	20	0.000187	1.18
Neisseria	*Neisseria polysaccharea*	2.46%	7	2.59E-05	1.18
Neisseria	*Neisseria sicca*	2.11%	6	3.95E-06	1.18
Neisseria	*Neisseria perflava*	2.11%	6	2.23E-05	1.18

Supplementary Table 2

Top five species in $COPSAC_{2000}$ from each of the genera found to be associated with asthma in the $COPSAC_{2010}$ cohort (Ref DOI: 10.1038/s41467-019-12989-7).
