# Supplementary material for: The airway microbiota of neonates colonized with asthma-associated pathogenic bacteria
Source: Nat Commun. 2023 Oct 20;14:6668. doi: 10.1038/s41467-023-42309-z (PMC10589220; doi:10.1038/s41467-023-42309-z)
Supplement: Supplementary file 3 — Reporting Summary [file 41467_2023_42309_MOESM3_ESM.pdf]

nature portfolio

Corresponding author(s): Jakob Stokholm

Last updated by author(s): Sep 27, 2023

Reporting Summary

Nature Portfolio wishes to improve the reproducibility of the work that we publish. This form provides structure for consistency and transparency in reporting. For further information on Nature Portfolio policies, see our Editorial Policies and the Editorial Policy Checklist.

Statistics

For all statistical analyses, confirm that the following items are present in the figure legend, table legend, main text, or Methods section.

n/a	Confirmed	
☐	☒	The exact sample size (*n*) for each experimental group/condition, given as a discrete number and unit of measurement
☐	☒	A statement on whether measurements were taken from distinct samples or whether the same sample was measured repeatedly
☐	☒	The statistical test(s) used AND whether they are one- or two-sided<br>*Only common tests should be described solely by name; describe more complex techniques in the Methods section.*
☐	☒	A description of all covariates tested
☐	☒	A description of any assumptions or corrections, such as tests of normality and adjustment for multiple comparisons
☐	☒	A full description of the statistical parameters including central tendency (e.g. means) or other basic estimates (e.g. regression coefficient) AND variation (e.g. standard deviation) or associated estimates of uncertainty (e.g. confidence intervals)
☐	☒	For null hypothesis testing, the test statistic (e.g. *F*, *t*, *r*) with confidence intervals, effect sizes, degrees of freedom and *P* value noted<br>*Give P values as exact values whenever suitable.*
☒	☐	For Bayesian analysis, information on the choice of priors and Markov chain Monte Carlo settings
☒	☐	For hierarchical and complex designs, identification of the appropriate level for tests and full reporting of outcomes
☐	☒	Estimates of effect sizes (e.g. Cohen's *d*, Pearson's *r*), indicating how they were calculated

Our web collection on statistics for biologists contains articles on many of the points above.

Software and code

Policy information about availability of computer code

Data collection	Clinical information on the cohort was collected during visits in a custom Oracle database on a Novel SQL server (Oracle Corp, Redwood Shores, CA)
Data analysis	Raw fastq files were demultiplexed using the Miseq Controller software prior to downstream analysis. The primers and adaptors in sequencing reads were removed using Cutadapt19. The determination of Amplicon Sequence Variants (ASVs) were performed on QIIME2 Core 2020.11 platform20 using Amplicon Denoising Algorithm 2 (DADA2) analysis pipelines21. The resulting ASV sequences were annotated using the AnnotIEM pipeline, which combines sequence alignment against four databases: EzBioCloud, NCBI, RDP, and Silva followed by a high confidence selection of best probable annotation.<br>To remove contamination from the human genome, we aligned representative ASV DNA sequences against the indexed reference human genome using Bowtie2 aligner in --very-sensitive mode and removed these mapped ASVs from the dataset.<br>Potential bacterial contaminants were removed using {decontam}<br>All statistical analyses were performed in R v. 4.2.1, and figures prepared using {ggplot2} v.3.3.6. Micro<br>biome data were analyzed using the package {phyloseq} v.1.40.0. Tables were prepared with {gtsummary} v.1.6.2.<br>Cox regression with log-scaled species relative abundances as predictors and DESeq2, adjusted for log(library size) and sequencing run.<br>Beta diversity (between-sample) was quantified using the weighted UniFrac metric and inference was calculated with the adonis2 PERMANOVA method from the R package {vegan} v.2.6-442.<br>We analyzed the V3-V4 resolution within the Moraxella genus using RibDif.<br>We compared co-occurrences of taxa with Spearman and SparCC70 correlations (as implemented in the R package {SpiecEasi} v. 1.1.2) and ordered axes using hierarchical clustering with the complete linkage method after converting correlation matrices to dissimilarity matrices with the function d = (1-c)/2. We obtained p-values for the SparCC correlations by bootstrapping with 1000 repeats.

No imputation of missing values was performed and a p-value below 0.05 was considered statistically significant throughout all analyses. All p-values were derived from two-sided tests. In the differential abundance analyses and pathogen score correlation taxa analyses, multiple comparisons were controlled using the Benjamini-Hochberg False Discovery Rate (FDR) correction71.
Please note that the AnnotIEM software package is under preparation for publication, and will be fully open access. Before such time, please contact Avidan Neumann (avidan.neumann@uni-a.de) for access.

For manuscripts utilizing custom algorithms or software that are central to the research but not yet described in published literature, software must be made available to editors and reviewers. We strongly encourage code deposition in a community repository (e.g. GitHub). See the Nature Portfolio guidelines for submitting code & software for further information.

Data

Policy information about availability of data

All manuscripts must include a data availability statement. This statement should provide the following information, where applicable:

- Accession codes, unique identifiers, or web links for publicly available datasets
- A description of any restrictions on data availability
- For clinical datasets or third party data, please ensure that the statement adheres to our policy

Source data are provided with this paper. The 16S sequencing data generated in this project is deposited in the European Nucleotide Archive (ENA) with the accession no PRJEB58215 (https://www.ebi.ac.uk/ena/browser/view/PRJEB58215) in an anonymized form. Individual-level personally identifiable clinical data from the children participating in the cohort cannot be made freely available, to protect the privacy of the participants and their families, in accordance with the Danish Data Protection Act and European Regulation 2016/679 of the European Parliament and of the Council (GDPR) that prohibit distribution even in pseudo-anonymized form. However, research collaborations are welcome, and data can be made available under a joint research collaboration by contacting the COPSAC Data Protection Officer (DPO), Ulrik Ralfkiaer, PhD (administration@dbac.dk).

Research involving human participants, their data, or biological material

Policy information about studies with human participants or human data. See also policy information about sex, gender (identity/presentation), and sexual orientation and race, ethnicity and racism.

Reporting on sex and gender	This study is a birth cohort study and both males and females were included. Information on sex composition of the cohort is available in table 1, and we was determined by parental interview and medical records. Sex-stratified results are included in the manuscript.
Reporting on race, ethnicity, or other socially relevant groupings	In table 1, we show the ethnicity composition. This information was obtained by parental interview. It is included since risk of childhood asthma may differ between between ethnicities. As can be seen in table 1, most participants are of northern european ethnicity. It is not used here as a proxy for socioeconomic status.
Population characteristics	The study included children from the COPSAC2000 cohort, which are 411 Danish neonates born to mothers with asthma, who were then followed prospectively through childhood and assessed for asthma up to age 7 years.
Recruitment	The Danish National Birth Cohort Study11 of approximately 100,000 pregnant women allowed targeted invitation of pregnant women. Women from the greater Copenhagen area who reported a history of asthma were invited to receive further information about the COPSAC. Also, we invited pregnant women with asthma who were attending prenatal clinics. Women who indicated an interest in the study were interviewed by telephone regarding eligibility criteria, including fluency in Danish, a physician's diagnosis of asthma after the age of 7 years, and a history of daily treatment with inhaled beta2-agonists or glucocorticoids for a minimum of 2 weeks during 2 seasons or continuously for 1 year. Eligible women subsequently received information by mail. Women reconfirming an interest were invited to the COPSAC Clinical Research Unit (CRU) for detailed information. Women who provided informed consent received guidance on cord blood sampling.<br>Since the cohort consists of children born to mothers with asthma, this may limit generalizability of our findings towards the background population, since they may display differences in etiological mechanisms of asthma.
Ethics oversight	The study was approved by approved by the Danish Local Ethics Committee (KF 01-289/96), and the Danish Data Protection Agency (2015-41-3696).

Note that full information on the approval of the study protocol must also be provided in the manuscript.

Field-specific reporting

Please select the one below that is the best fit for your research. If you are not sure, read the appropriate sections before making your selection.

[x] Life sciences [ ] Behavioural & social sciences [ ] Ecological, evolutionary & environmental sciences

For a reference copy of the document with all sections, see nature.com/documents/nr-reporting-summary-flat.pdf

Life sciences study design

All studies must disclose on these points even when the disclosure is negative.

Sample size	Sample size was limited by the number of participants in the cohort, we used all available samples in this study

Data exclusions	Infants of consenting mothers were enrolled in the cohort at the CRU visit 1 month after birth, excluding infants with a severe congenital anomaly, a gestational age younger than 36 weeks, a need for mechanical ventilation, or a lower respiratory tract infection.
Replication	We compared the findings between two cohorts, which did not replicate but showed similar findings, please refer to fig 4. The cohort was not otherwise replicated.
Randomization	There was no randomization in the study - the study is an observational cohort study of children, not an intervention study.
Blinding	There was no blinding in the study - the study is an observational cohort study with no intervention that could be blinded.

Reporting for specific materials, systems and methods

We require information from authors about some types of materials, experimental systems and methods used in many studies. Here, indicate whether each material, system or method listed is relevant to your study. If you are not sure if a list item applies to your research, read the appropriate section before selecting a response.

Materials & experimental systems

n/a	Involved in the study
☒	☐ Antibodies
☒	☐ Eukaryotic cell lines
☒	☐ Palaeontology and archaeology
☒	☐ Animals and other organisms
☒	☐ Clinical data
☒	☐ Dual use research of concern
☒	☐ Plants

Methods

n/a	Involved in the study
☒	☐ ChIP-seq
☒	☐ Flow cytometry
☒	☐ MRI-based neuroimaging
